# Corn Seed Defect Detection Based on Watershed Algorithm and Two-Pathway Convolutional Neural Networks

**DOI:** 10.3389/fpls.2022.730190

**Published:** 2022-02-23

**Authors:** Linbai Wang, Jingyan Liu, Jun Zhang, Jing Wang, Xiaofei Fan

**Affiliations:** ^1^State Key Laboratory of North China Crop Improvement and Regulation, Hebei Agricultural University, Baoding, China; ^2^College of Mechanical and Electrical Engineering, Hebei Agricultural University, Baoding, China

**Keywords:** corn seed defect, multispectral image, object detection, watershed segmentation algorithm, convolutional neural network

## Abstract

Corn seed materials of different quality were imaged, and a method for defect detection was developed based on a watershed algorithm combined with a two-pathway convolutional neural network (CNN) model. In this study, RGB and near-infrared (NIR) images were acquired with a multispectral camera to train the model, which was proved to be effective in identifying defective seeds and defect-free seeds, with an averaged accuracy of 95.63%, an averaged recall rate of 95.29%, and an F1 (harmonic average evaluation) of 95.46%. Our proposed method was superior to the traditional method that employs a one-pathway CNN with 3-channel RGB images. At the same time, the influence of different parameter settings on the model training was studied. Finally, the application of the object detection method in corn seed defect detection, which may provide an effective tool for high-throughput quality control of corn seeds, was discussed.

## Introduction

Corn is one of the most important crops in the world (Afzal et al., 2017), which is widely planted around the Earth. Its output and trade volume have kept increasing in recent years. In the process of circulation, appearance quality is a critical factor that influences corn seed price. Corn seeds are vulnerable to damage and mildew during storage and transportation, and phenotypic defect is an important index of seed quality evaluation. At present, seed quality detection still relies on the method of traditional manual identification, which employs low efficiency and strong subjectivity. With the development of computer vision technology (Rehman et al., 2018; Gutiérrez et al., 2019; Keiichi et al., 2019; Azimi et al., 2020; Arunachalam and Andreasson, 2021), image processing methods based on machine learning are applied to seed quality classification and have achieved good results. Kiratiratanapruk and Sinthupinyo (2012) proposed a method to classify more than 10 levels of seed quality by using color and texture features with a support vector machine (SVM) classifier. Ke-Ling et al. (2018) proposed a method of high-quality pepper seed screening based on machine vision, which could be used to predict the germination rate of seeds effectively, and therefore provided a guide for seed quality selection. Ali et al. (2020) discussed the feasibility of the machine learning method in corn seed classification. While the traditional machine learning methods normally require extracting the features manually, which are usually not comprehensive enough, the recognition accuracy, therefore, is limited.

In recent years, as a representative of deep learning technology, convolutional neural networks (CNNs) develop rapidly and are widely used for image recognition (Afonso et al., 2019; Altuntaş et al., 2019; Gao et al., 2020; Zhang C. et al., 2020). Compared with traditional machine learning technology, CNNs are naturally embedded with a feature learning part through the combination of low-level features to form more abstract high-level features. Many researchers have applied CNNs to the field of agriculture. Laabassi et al. (2021) proposed a CNN model to classify wheat varieties, and the accuracy of classification was between 85.00 and 95.68%. Pang et al. (2020) developed a method for rapid estimation and prediction of corn seed vigor using a hyperspectral imaging system with deep learning. The recognition accuracy of the 1D-CNN model reached 90.11%, and the recognition accuracy of the 2D-CNN model reached 99.96%. Sj et al. (2021) proposed a method to extract the characteristics of corn seeds by using a deep CNN and then classifying the varieties. The results showed that CNNs were effective in corn seed classification.

In this article, RGB and NIR images (Kusumaningrum et al., 2018) collected by a multispectral camera were used to train a CNN model. To solve the problem of corn seed adhesion and seed location during the recognition process, a watershed algorithm (Lei et al., 2019; Sta et al., 2019; Zhang et al., 2021) combined with a two-way CNN (Zhang J. J. et al., 2020) was proposed to detect corn seed defects. The results revealed that this method is with high accuracy, and the targets can be accurately located and classified. This method may provide a theoretical basis for the subsequent development of a seed quality control device.

## Materials and Methods

### Experimental Material and Instruments

**FIGURE 1:**
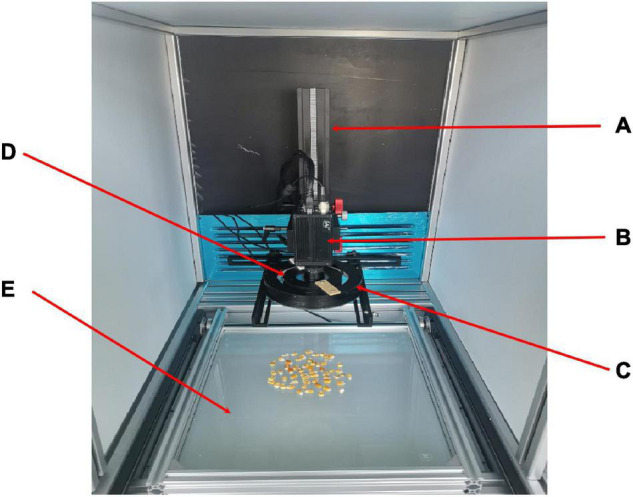
Image acquisition platform. **(A)** Support, **(B)** camera, **(C)** circular white light source, **(D)** circular near-infrared light source, and **(E)** white backlight.

**FIGURE 2:**
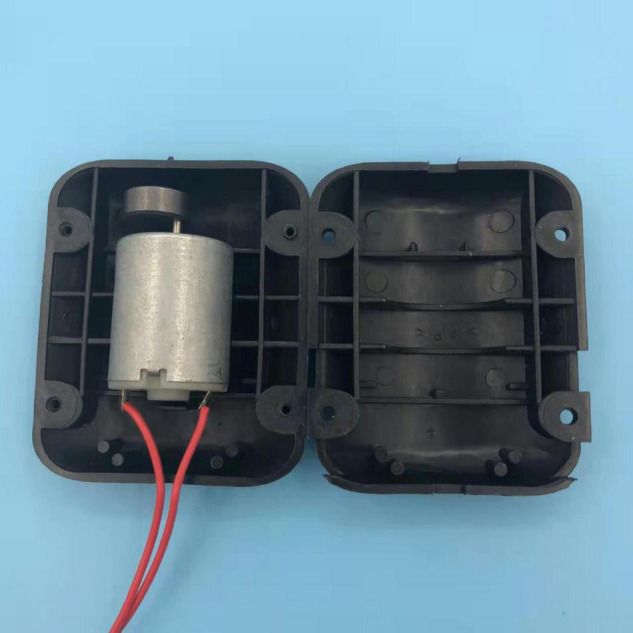
The vibration module.

In this experiment, 2,365 corn seeds from three different varieties (Zhengdan 985, Keshi 982, Jiyu 517) were adopted as experimental materials. Some seeds were defect-free in appearance, and the other seeds were with defects, including mold, insect or mechanical damages, and discoloration. A 4-channel (RGB + NIR) multispectral camera (LQ-200CL, JAI, Denmark) was used for image acquisition, with 8 bits for each channel and a resolution of 1,296 * 964. A white LED ring light, coupled with a near-infrared ring light, and a white backlight panel were used to enhance the image contrast. The image acquisition platform is shown in Figure 1. At the same time, to prevent the seeds from overlapping, the vibration module was placed under the backlight panel (shown in Figure 2). The motor voltage is 12 V. The rotational speed of the motor is 8,000 rpm. The size of the vibrating head is 3.5 cm. It is found that the vibration module shows a very good effect in restraining seed overlap and shielding.

The experiment was based on Windows 10, a 64-bit operating system with CUDA 10.0, and python programming language, along with TensorFlow and Keras deep learning framework. The computer used for the experiment employed a GeForce GTX 1660 graphics card, with 6G memory, and an Intel (R) Core (TM) i5-9400f processor with the main frequency of 2.90 GHz.

### Data Acquisition

**FIGURE 3:**
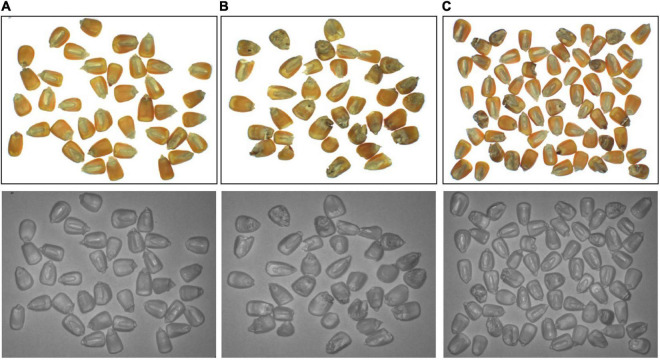
The original image of **(A)** good quality corn seeds, **(B)** disfigured corn seeds, and **(C)** both situations.

A total of 50 samples of corn seed with no defects (1,066 single seeds overall) and fifty seed samples with different appearance defects (1,042 single seeds overall) were imaged. The images of another 10 samples with both defective and defect-free seeds were also acquired for the verification of the final model, with an overall 257 single seeds. Each sample was captured in one image deck, which contained RGB and NIR images, with a size of 1,296 × 964. The images acquired are shown in Figure 3. To solve the issue of adhesion among seeds in the images, a watershed algorithm was applied to each image, and all individual seeds were segmented. Eventually, each seed was extracted from the original image to form a new image, which was resized to 224 × 224 with bilinear interpolation.

To improve the performance of the model, data augmentation was implemented for image decks of individual seeds. The enhancement methods (Huang et al., 2019; Tiwari et al., 2021) included brightness adjustment, rotation, applying Gaussian noise, etc. The images of defect-free seeds were labeled as “good,” and the images of defective seeds were labeled as “bad.” Eventually, there were 3,913 images (RGB + NIR) of defect-free seeds and 3,913 images of defective seeds, respectively. The training set and the testing set were divided by 4:1, and therefore, 5,869 images with single seed were used for training, and 1,957 images were used for testing.

### Watershed Algorithm

**FIGURE 4:**
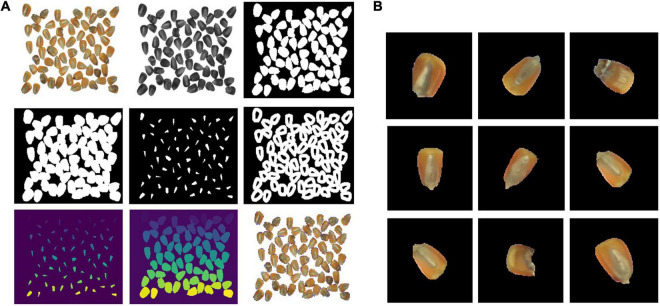
Image processing procedures. **(A)** Segmentation processes and **(B)** segmentation results.

Every single seed in the image deck was segmented using the watershed algorithm. First, the original 4-channel image was converted to a grayscale image. By comparing the four layers (R, G, B, and NIR), the results showed that the B-channel image was the best to use for binarization. Binarization was then performed, and any noise in the binary image was removed by a morphological open operation. An expansion operation was then applied to the binary image, and a distance transforming algorithm was used to obtain the central region of each seed. The edge of the seed was the dilational image subtracted from the central regions. The central region of each seed was then naturally separated from each other. Finally, the watershed algorithm was used to extract the edge of the seeds, and each seed was segmented in the image by position coordinates. The segmentation processes are shown in Figure 4. The NIR images were then segmented using the position coordinates from the segmented RGB images. The combination of the RGB image and NIR image of each seed was used for training or detection processes.

### Corn-Seed-Net Model Structure

**FIGURE 5:**
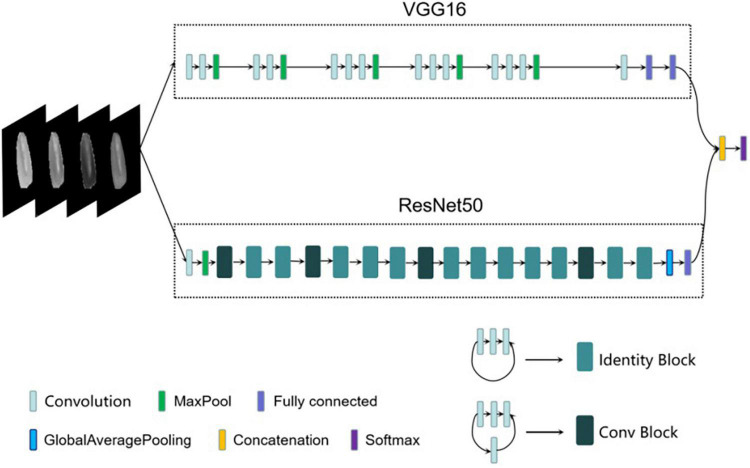
Corn-seed-Net network architecture.

Every single seed was separated by the watershed algorithm, and the position coordinates were obtained. The CNN model was then used to detect the quality of the corn seeds. The detection results were marked in the image according to the position coordinates. In this article, a two-pathway CNN, Corn-seed-Net (shown as Figure 5), was designed combining VGG16 (Simonyan and Zisserman, 2015) and ResNet50 (He et al., 2016). The model was used to extract deep features of 4-channel corn seed images and then classify them.

To reduce the number of parameters, continuous convolution kernels of 3 × 3 were used in the VGG16. Thirteen convolution layers were used to extract deeper image features and increase the fitting capacity and the expressive capacity of the model. However, as the number of network layers increases, the gradient of the model disappears or explodes, which makes the performance of the model plummet. However, the residual structure was added in the ResNet50, the input of the convolution layer was directly added to the output of the convolution layer, and it solves the degradation problem of deep CNN. Therefore, the advantages of both VGG16 and ResNet50 were combined in the Corn-seed-Net.

In this article, the VGG16 branch was optimized. The number of parameters of the last two fully connected layers of the original models was tremendous. To avoid feature information redundancy, a convolution layer of 7 × 7 was applied to the final max-pooling layer, with 512 channels, and two fully connected layers composed of 512 feature vectors were added. In this way, the number of parameters was reduced. For the ResNet50 branch, after the global average pooling layer, a fully connected layer composed of 512 feature vectors was added. The two branches were then fused with the final fully connected layer, and the vectors of the generated features were 1,024. Finally, the classification was completed through the Softmax layer, with the category number set to 2.

The Softmax function was used to calculate the probability of classification, and the calculation formula is as follows:


(1) $$y_{i\,m}=\frac{e^{z_{i\,m}}}{\sum_{k=1}^{k}e^{z_{i\,k}}}$$

In the formula, *y*_*im*_ is the prediction probability that the *i*th sample belongs to class *m*, *k* is the number of categories, *z*_*im*_ is the product of the output vector of the *i*th sample and the parameter vector of class *m*, and *z*_*ik*_ is the product of the output vector of the *i*th sample and the parameter vector of class *k.*

Categorical cross-entropy was used to calculate the loss function of the model, and the formula is given as follows:


(2) $$L=-\sum_{i=1}^{n}{\hat{y}}_{i\,m}\,\mathrm{lg}y_{i\,m}$$

In the formula, *L* is the loss function, *n* is the number of images in each batch, and *y*_*im*_ is the expected probability that the *i*th sample belongs to class *m*.

### Parameter Set

To achieve the best result, the model was trained by setting different parameters. The momentum used in the final model was 0.9, and the initial value of the learning rate was set as 0.001. Stochastic gradient descent (SGD) (Samik and Sukhendu, 2018) algorithm was used with 100 epochs for the training. In the process of training, when the loss of the test set no longer decreased, the learning rate was reduced by half. Other parameters were set to default. The accuracy of the final training set was 100.00%, and the accuracy of the test set was 96.90%.

### Hand-Crafted Feature Extraction

In this article, five hand-crafted feature extraction methods were used to extract the features of a single seed segmented by watershed algorithm, and then an SVM classifier was used for seed classification. The feature extraction methods were as follows:

(1) Morphological characteristics (MC) (Zhang L. et al., 2020) were used to binarize each seed’s image. The ratio of the perimeter of the seed area, the diameter of the circle with the same area, the eccentricity of the fitted ellipse, the ratio of the major axis to minor axis, and the ratio of area to bounding box area from a connected domain were then extracted. A total of five morphological features were used as feature vectors.
(2) Color features have little dependence on image size and position. In this article, the parameters related to color (RGB) histogram were extracted as feature vectors.
(3) Local shape information can be well captured by histogram of gradient (HOG) (Dalal, 2005), and it is relatively stable to the change of geometry and optics. In this article, the gradient information of the image was extracted as feature vectors.
(4) Gray-level co-occurrence matrix (GLCM) (Haralick et al., 1973) is a method of texture feature extraction based on statistics. The statistics constructed in this article include contrast, dissimilarity, homogeneity, energy, correlation, and angular second moment. These six characteristic parameters were used as feature vectors.
(5) Local binary pattern (LBP) (Ojala et al., 2002) features have the advantages of gray invariance and rotation invariant. In this article, the LBP value of the image was extracted and used to represent the texture information of the region. Finally, the statistical histogram of LBP features was used as the feature vectors.

### Evaluation Index

In this article, to evaluate the accuracy and stability of the training model for seed quality identification, the precision and recall ratios were used to evaluate the model, and the *F*_1_ value was used as the average evaluation of them. The evaluation formulas are given as follows:


(3) $$p=\frac{n\,\text{TP}}{n\,\text{TP}+n\,\text{TF}}\times 100\%$$

(4) $$R=\frac{n\,\text{TP}}{n\,\text{TP}+n\,\text{FN}}\times 100\%$$

(5) $$F\,1=\frac{2\,P\,R}{P+R}\times 100\%$$

where *n*_*TP*_ is the number of corn seeds correctly identified, *n*_*FP*_ is the number of misidentified corn seeds, and *n*_*FN*_ is the number of unrecognized corn seeds.

## Results and Discussion

### The Selection of Corn-Seed-Net Model Structure

**FIGURE 6:**
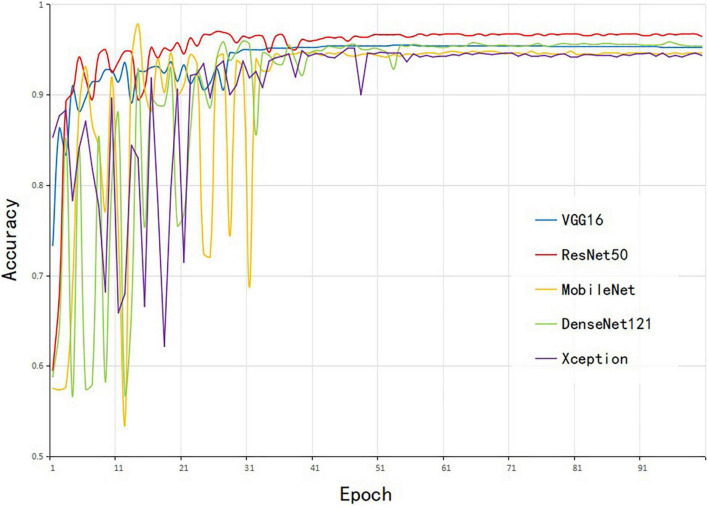
The accuracy of the five models for the test set.

To select an optimal model structure, the 4-channel images (RGB + NIR) were used for training five CNN models [e.g., VGG16, ResNet50, MobileNet, DenseNet121 (Huang et al., 2016), and Xception (Chollet, 2017)]. The weights trained on the ImageNet dataset were used for parameter initialization, and the same dataset was trained for the model; the accuracy of the test set is shown in Figure 6. The number of input channels for the model was set to 4, but the number of convolution layers was unchanged. The number of parameters for convolutional layers was the same as the original model, and therefore, the pre-trained weights from ImageNet were used in this study. The five models converged after 50 epochs, and the accuracy stabilized at a high value. For the five models, the ResNet50 model had the highest accuracy of 96.63%. The VGG16 model converged most rapidly. The DenseNet model achieved the characteristics of dense connection through repeated splicing, but the running memory consumption was large and the convergence time was long. The MobileNet model possessed a smaller amount of parameters but the accuracy was low. Deep separable convolution and residual connection were used in the Xception model, and the accuracy was also relatively low. Considering the accuracy and convergence, VGG16 and ResNet50 were combined to construct the final model.

### The Selection of Corn-Seed-Net Model Parameters

**FIGURE 7:**
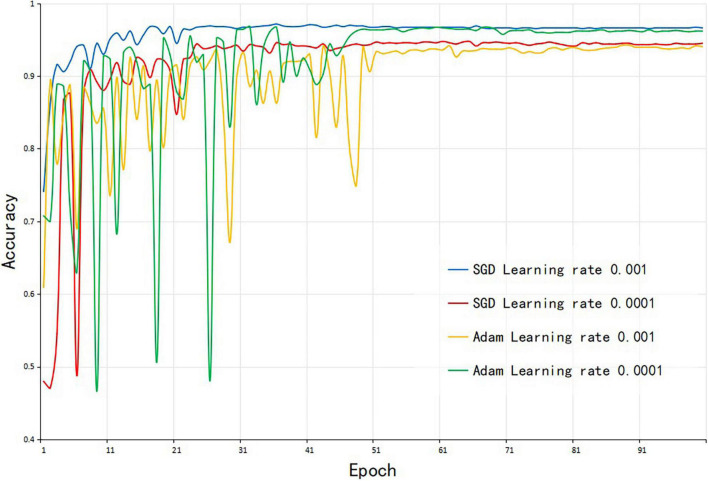
The accuracy of Corn-seed-Net with different model parameters.

To obtain faster training speed and better convergence performance of the model, the same 4-channel images were used to train the model, and two branches of Corn-seed-Net were initialized with the weights trained using the ImageNet dataset. The influences of different initial learning rates and different optimization algorithms on the model were tested (as shown in Table 1). Figure 7 shows that the SGD algorithm converged faster, and the Adam algorithm was unstable in the first half of the training process. Therefore, the SGD optimization algorithm was used in the experiment, and the initial learning rate was set to 0.001.

**TABLE 1:** Training results of Corn-seed-Net with different model parameters.

Initial learning rate	Training algorithm	Epoch time/s	Training accuracy/%	Validation accuracy/%
0.001	Adam	180	100.00	94.23
0.0001	Adam	180	100.00	95.80
0.001	SGD	165	100.00	96.90
0.0001	SGD	165	99.98	94.59

### Test Results of the Corn-Seed-Net Model on a Single Seed

To verify the classification accuracy of the Corn-seed-Net model for 4-channel images of a single seed, 100 corn seeds without any defects in appearance and another 100 seeds with defected appearance were selected in this experiment. At the same time, other one-pathway CNN models were compared, and the results are shown in Table 2. The averaged accuracy of the Corn-seed-Net model for each single seed classification is up to 100%, which was better than other one-pathway models. The averaged detection time for a single seed was 68 ms, which indicated that the two-pathway model is suitable for seed classification.

**TABLE 2:** Test results of single seed with different models.

Model	Classes	Predict classes		Model performance		
		Good	Bad	Accuracy/%	Averaged accuracy/%	Detection time/ms
VGG16	Good	99	1	99	99.00	45.5
	Bad	1	99	99		
ResNet50	Good	100	0	100	99.00	41.7
	Bad	2	98	98		
MobeliNet	Good	97	3	97	98.00	22.9
	Bad	1	99	99		
DenseNet121	Good	97	3	97	97.50	58.0
	Bad	2	98	98		
Xception	Good	98	2	98	97.50	41.0
	Bad	3	97	97		
Corn-seed-Net	Good	100	0	100	100.00	68.0
	Bad	0	100	100		

### Detection Results of the Corn-Seed-Net Model Combined With the Watershed Algorithm

**FIGURE 8:**
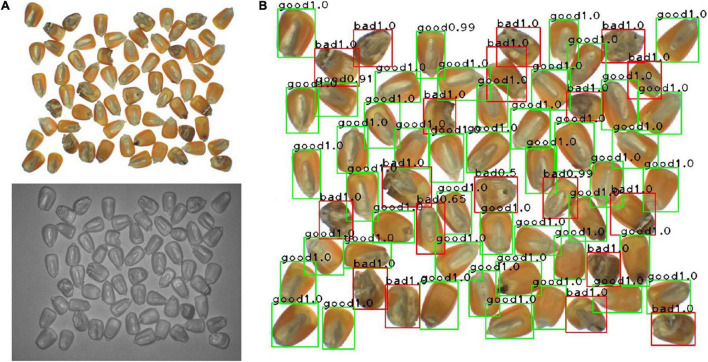
**(A)** The original image and **(B)** object detection results.

To accurately locate each seed with the quality rating, the watershed algorithm was adopted and combined with the Corn-seed-Net model on 4-channel images of corn seed (Figure 8A). The conglutinated seeds were segmented using the watershed algorithm, and meanwhile, the position coordinates of each seed were also obtained. The detection results are shown in Figure 8B.

To evaluate the performance of this method, 10 groups of images were used for verification. At the same time, other one-pathway CNNs were compared, and the results are shown in Table 3. It showed that the watershed algorithm combined with the Corn-seed-Net model had the highest precision and recall rate on average, and the F1 value was 95.46%. Due to the addition of the operation of the watershed segmentation during image detection, there was an increase in the detection time, and the averaged detection time for a single seed was 149.55 ms. The results appeared that the model performance improves when the watershed algorithm was adopted and combined with two-pathway CNN.

**TABLE 3:** Comparison of model performance combined with watershed algorithm.

Model	Classes	Precision/%	Recall/%	Averaged precision/%	Averaged recall/%	F1/%	Detection time/ms
VGG16	Good	90.91	96.55	93.05	94.71	93.87	139.5
	Bad	95.19	92.86				
ResNet50	Good	94.00	97.24	94.69	94.60	94.64	122.5
	Bad	95.37	91.96				
MobeliNet	Good	93.85	94.48	91.96	93.22	92.59	95.95
	Bad	91.96	91.96				
DenseNet121	Good	91.45	95.86	92.43	92.13	92.27	136.75
	Bad	93.40	88.39				
Xception	Good	94.48	94.48	93.26	93.67	93.46	133.85
	Bad	92.03	92.86				
Corn-seed-Net	good	94.08	98.62	95.63	95.29	95.46	149.55
	bad	97.17	91.96				

### RGB Images Detection Results

To investigate whether using 4-channel images (RGB + NIR) is superior to 3-channel images (RGB) in seed classification, RGB images of the same dataset were used in the experiment, with watershed algorithm combined with Corn-seed-Net model, and the results are shown in Table 4. It is shown that the extra information carried with the NIR band improved the model performance on both precision and recall rate, compared with the models obtained with RGB images only.

**TABLE 4:** Comparison of model performance combined with watershed algorithm using RGB images.

Model	Classes	Precision/%	Recall/%	Averaged precision/%	Averaged recall/%	F1/%	Detection time/ms
RGB VGG16	Good	90.13	94.48	91.67	90.10	90.87	69.92
	Bad	93.20	85.71				
RGB ResNet50	Good	95.56	95.86	94.54	93.02	93.77	98.83
	Bad	93.52	90.18				
RGB Corn-seed-Net	Good	93.33	96.55	93.80	92.47	93.13	117.63
	Bad	94.28	88.39				
RGB + NIR Corn-seed-Net	Good	94.08	98.62	95.63	95.29	95.46	149.55
	Bad	97.17	91.96				

To fully evaluate the performance of the watershed algorithm combined with the Corn-seed-Net model, the watershed algorithm combined with the traditional feature extraction method was also studied, and SVM was used to classify the corn seeds based on their quality. The results are shown in Table 5, and it indicates that the precision, recall, and F1 of the method we have proposed were all significantly higher than those of the traditional methods, as more deep image features were extracted in CNN.

**TABLE 5:** Comparison of object detection results.

Model	Average precision/%	Average recall/%	F1/%
GLCM + SVM	22.05	48.11	30.24
Color + SVM	60.97	58.74	59.83
HOG + SVM	64.30	64.07	64.18
MC + SVM	68.64	68.17	68.40
LBP + SVM	74.28	73.73	74.00
Corn-seed-Net	95.63	95.29	95.46

## Discussion

At present, some studies have been devised in seed classification using imaging technology combined with machine learning and deep learning (Huang et al., 2019; Kozowski et al., 2019; Ansari et al., 2021). However, most of the studies were based on RGB imaging technology rather than using four-channel multispectral images. Moreover, there are few studies on seed quality detection using the current typical object detection algorithm. This article designed an end-to-end object detection model, and high accuracy was achieved in seed quality detection.

In this article, RGB and NIR images of corn seeds were obtained using a multispectral camera, and the watershed algorithm combined with the Corn-seed-Net model was used to predict the quality of corn seeds. The watershed algorithm is used to segment every single seed and obtain the precise location of the seed. At the same time, while the 4-channel image data with both RGB and NIR bands were used as the inputs of the Corn-seed-Net model, the accuracy of the model was better than that with RGB images only.

The Corn-seed-Net model combines the advantages of VGG16 and ResNet50, and deeper information could be extracted by deep networks. It employs a residual network structure, and the effect of degradation of the deep network is eliminated. With the optimized model, 200 single corn seeds were used for verification and compared with other single-pathway models, and the results revealed that the average classification accuracy of the Corn-seed-Net model reached 100.00%.

To evaluate the corn seed defect detection performance of the watershed algorithm combined with the Corn-seed-Net model, we compared the detection results with RGB images and traditional feature extraction methods. The experimental results showed that the proposed method in this article had the best performance, with an average precision of 95.63%, an average recall rate of 95.29%, and an F1 value of 95.46%.

## Conclusion

In this study, an end-to-end corn seed object detection model was proposed, which combined watershed segmentation algorithm and CNNs. In comparison with mainstream object detection models (e.g., Faster-RCNN, SSD, and YOLO), our method uses a watershed segmentation algorithm to obtain more accurate target positions, which also reduces the complexity of the network at the same time. In addition, this method eliminates the manual annotation of the image and reduces the workload of dataset preparation. In the future, this method can be further optimized by simplifying the network structure, which may shorten the calculation time while ensuring the classification accuracy, to provide a basis for the subsequent development of a quality detection device.

## Data Availability Statement

The raw data supporting the conclusions of this article will be made available by the authors, without undue reservation.

## Author Contributions

LW, JL, and XF conceived the idea, proposed the method, and revised the manuscript. LW, JW, and JZ contributed to the preparation of equipment and acquisition of data, wrote the code, and tested the method. LW, JZ, and JL contributed to the validation results. LW and XF wrote the manuscript. All authors read and approved the final manuscript.

## Conflict of Interest

The authors declare that the research was conducted in the absence of any commercial or financial relationships that could be construed as a potential conflict of interest.

## Publisher’s Note

All claims expressed in this article are solely those of the authors and do not necessarily represent those of their affiliated organizations, or those of the publisher, the editors and the reviewers. Any product that may be evaluated in this article, or claim that may be made by its manufacturer, is not guaranteed or endorsed by the publisher.
